# Supplementary material for: The Systems Biology Research Tool: evolvable open-source software
Source: BMC Syst Biol. 2008 Jun 29;2:55. doi: 10.1186/1752-0509-2-55 (PMC2446383; doi:10.1186/1752-0509-2-55)
Supplement: Additional file 1 — SBRT Archive. An archive of the current version of the Systems Biology Research Tool. [file 1752-0509-2-55-S1.zip › sbrt-1.4.0/doc/developers_guide/api/sbrt/kernel/math/class-use/HashLinearComb.html]

Uses of Class sbrt.kernel.math.HashLinearComb


|  |  |  |
| --- | --- | --- |
| **Overview** \| **Package** \| **Class** \| **Use** \| **Tree** \| **Deprecated** \| **Index** \| **Help** | |  |
| PREV   NEXT | **FRAMES**    **NO FRAMES**     **All Classes** |  |


---


## **Uses of Class sbrt.kernel.math.HashLinearComb**

| Packages that use HashLinearComb | |
| --- | --- |
| **sbrt.kernel.math** | Provides classes and interfaces for creating objects relevant to mathematics. |

Uses of HashLinearComb in sbrt.kernel.math

| Methods in sbrt.kernel.math that return HashLinearComb | |
| --- | --- |
| `HashLinearComb<V>` | `HashLinearComb.add(double constant)`             Adds, in the mathematical sense, the provided constant to this linear combination. |
| `HashLinearComb<V>` | `HashLinearComb.add(LinearComb<? extends V> expression)`             Adds, in the mathematical sense, the provided linear combination to this linear combination. |
| `HashLinearComb<V>` | `HashLinearComb.multiply(double constant)`             Multiplies this linear combination by the provided constant. |
| `HashLinearComb<V>` | `HashLinearComb.plugIn(java.util.Map<?,? extends LinearComb<? extends V>> variableValues)`             Plugs in the provided linear combinations to produce a new linear combination. |
| `HashLinearComb<V>` | `HashLinearComb.plugIn(java.util.Map<?,java.lang.Double> numbers, java.util.Map<?,? extends LinearComb<? extends V>> linearCombs)`             Plugs in the provided numerical values and linear combinations to produce a new linear combination. |
| `HashLinearComb<V>` | `HashLinearComb.plugIn(java.lang.Object variable, double value)`             Plugs the provided numerical value into the provided variable to produce a new linear combination. |
| `HashLinearComb<V>` | `HashLinearComb.plugIn(java.lang.Object variable, LinearComb<? extends V> expr)`             Plugs the provided linear combination into the provided variable to produce a new linear combination. |
| `HashLinearComb<V>` | `HashLinearComb.plugInNumbers(java.util.Map<?,java.lang.Double> variableValues)`             Plugs in the provided numerical values to produce a new linear combination. |
| `static <V> HashLinearComb<V>` | `HashLinearComb.singleTerm(double coefficient, V variable)`             Returns a single-term linear combination with the provided variable and coefficient. |
| `static <V> HashLinearComb<V>` | `HashLinearComb.singleTerm(LinearComb.Term<V> term)`             Returns a single-term linear combination with the provided term. |
| `static <V> HashLinearComb<V>` | `HashLinearComb.singleTerm(V variable)`             Returns a single-term linear combination with the provided variable. |
| `static <V> HashLinearComb<V>` | `HashLinearComb.singleTerm(V variable, double coefficient)`             Returns a single-term linear combination with the provided variable and coefficient. |
| `static <V> HashLinearComb<V>` | `HashLinearComb.singleTerm(V variable, double coefficient, double zeroCutoff)`             Returns a single-term linear combination with the provided variable, coefficient, and zero cutoff. |
| `HashLinearComb<V>` | `HashLinearComb.solveFor(java.lang.Object variable, double rightHandSide)`             Equates this linear combination to the provided constant and solves for the provided variable. |

---


|  |  |  |
| --- | --- | --- |
| **Overview** \| **Package** \| **Class** \| **Use** \| **Tree** \| **Deprecated** \| **Index** \| **Help** | |  |
| PREV   NEXT | **FRAMES**    **NO FRAMES**     **All Classes** |  |


---
